# Auditory distraction during reading: Investigating the effects of background sounds on parafoveal processing

**DOI:** 10.1177/17470218241269327

**Published:** 2024-08-28

**Authors:** Laura Rettie, John E Marsh, Simon P Liversedge, Mengsi Wang, Federica Degno

**Affiliations:** 1 School of Psychology and Humanities, University of Central Lancashire, Preston, UK; 2 Engineering Psychology, Humans and Technology, Department of Business Administration, Technology and Social Sciences, Luleå University of Technology, Luleå, Sweden; 3 Faculty of Psychology, Tianjin Normal University, Tianjin, China; 4 Department of Psychology, Bournemouth University, Poole, UK

**Keywords:** Auditory distraction, background sound, auditory deviant effect, reading, eye movements

## Abstract

Previous research suggests that unexpected (deviant) sounds negatively affect reading performance by inhibiting saccadic planning, which models of reading agree takes place simultaneous to parafoveal processing. This study examined the effect of deviant sounds on foveal and parafoveal processing. Participants read single sentences in quiet, standard (repeated sounds), or deviant sound conditions (a new sound within a repeated sound sequence). Sounds were presented with a variable delay coincident with the onset of fixations on target words during a period when saccadic programming and parafoveal processing occurred. We used the moving window paradigm to manipulate the amount of information readers could extract from the parafovea (the entire sentence or a 13-character window of text). Global, sentence-level analyses showed typical disruption to reading by the window, and under quiet conditions similar effects were observed at the target and post-target word in the local analyses. Standard and deviant sounds also produced clear distraction effects of differing magnitudes at the target and post-target words, though at both regions, these effects were qualified by interactions. Effects at the target word suggested that with sounds, readers engaged in less effective parafoveal processing than under quiet. Similar patterns of effects due to standard and deviant sounds, each with a different time course, occurred at the post-target word. We conclude that distraction via auditory deviation causes disruption to parafoveal processing during reading, with such effects being modulated by the degree to which a sound’s characteristics are more or less unique.

Coherent and efficient mental performance critically depends on the ability to focus on incoming inputs relevant to current goals while ignoring task-irrelevant stimulation (e.g., Cowan, 1998; Engle, 2002). Nonetheless, the selective attention system is permeable to the processing of task-irrelevant stimulation, allowing an individual to detect, and when necessary act upon, potentially important changes in their environment (Allport, 1993). This study aims to shed light on the mechanisms through which auditory stimuli affect reading, a skill that plays an important role in many daily tasks.

A large body of research has investigated the effect of background sounds on task performance (for reviews, see Dalton & Behm, 2007; Klatte et al., 2013; Szalma & Hancock, 2011). These studies have shown that little or no disruption occurs when a repeated, task-irrelevant (standard) sound is presented (e.g., Campbell et al., 2002; Jones et al., 1992; Jones & Macken, 1993). However, performance is impaired when a change occurs in the auditory stream. A single change (i.e., a deviant) presented in a standard, steady-state sequence of sounds (e.g., AAABA^1^) disrupts performance on a variety of attentionally demanding serial and non-serial tasks when compared with standard, steady-state sequences, as seen for example in missing item, oddball, and serial short-term memory tasks (i.e., the *auditory deviant effect*; Hughes et al., 2005; Lange, 2005).Furthermore, a sound sequence conveying appreciable change from one item to the next (e.g., ABABA), as compared with a steady-state sequence of sounds, produces pronounced disruption to tasks involving short-term maintenance of serial order (i.e., the *changing-state effect*; e.g., Jones et al., 1992).

The auditory deviant effect has been typically revealed in the context of cross-modal oddball (e.g., Escera et al., 1998, 2002) and short-term memory tasks (e.g., Hughes et al., 2005, 2007). In those settings, research has shown that unexpected deviants cause delayed reaction times or increased error rates when categorising or recalling visual stimuli respectively (e.g., Escera et al., 1998, 2002; Hughes et al., 2005, 2007). Disruption due to the presence of a deviant auditory distractor embedded in a repeated sequence of sounds (standards) is suggested to arise because deviants violate the existing mental representation of the repeated sounds: a representation which specifies, among other features, that each sound occurs with equal temporal regularity and is the same as its predecessor (e.g., Hughes et al., 2005; Parmentier et al., 2011; Sokolov, 1963). When violations to that mental representation are present, an involuntary orienting of attention towards the deviant sound occurs, which causes some temporary withdrawal of resources from the focal task (*call for attention;* e.g., Bell et al., 2010, 2019; Campbell et al., 2003; Escera et al., 2000) and attention shifts towards the unexpected sound (*attentional capture*; Bell et al., 2019; Cowan, 1998; Elliott, 2002; Hughes, 2014; Schröger, 1997).

The existing accounts that explain the mechanisms by which auditory stimuli affect performance agree that deviant sounds cause attentional capture. However, whether other types of background sound (e.g., standard or changing-state sequences) give rise to attentional capture is a matter of debate. The Duplex account (Hughes, 2014; Hughes et al., 2005, 2007) suggests that although both deviant and changing-state sound sequences result in greater disruption than standard sound sequences, which cause little or no disruption, only deviant sounds produce attentional capture. The so-called Unitary account^2^ (Bell et al., 2010, 2012; Cowan, 2001), as coined by Hughes et al. (2007), however, suggests that both deviant and changing-state, but not standard, sound sequences result in attentional capture. Yet, there also exists a body of research providing evidence for an alternative “graded” attentional capture account (Bell et al., 2019; Röer et al., 2014a; Schröger et al., 2000). According to this view, all sounds (whether standard, deviant, or changing-state) produce a call for attention. However, disruption to focal task performance is graded, depending on whether or not the current sound matches the existing mental representation of the previous sound. For standard, steady-state sequences, there is a small amount of disruption relative to quiet, due to the temporary withdrawal of resources from the focal task (i.e., call for attention), but attentional capture does not occur, as the call for attention is denied since the current sound matches the mental representation of its predecessor (Bell et al., 2019). In addition, standard, steady-state sounds produce less disruption than changing-state and deviant sounds (e.g., Jones & Macken, 1993), as the latter are incongruent with the mental representation and thus both call for attention and subsequent attentional capture takes place.

Neuroimaging research has provided further support for the notion of attentional capture by deviants. This research has shown that the orienting of attention towards the deviant sound is controlled by two neural systems which interact during task completion to determine what we attend to (e.g., Corbetta et al., 2000; Corbetta & Shulman, 2002 for a review, see Corbetta et al., 2008). The first of these is a dorsal, goal-directed system which is responsive to stimuli relevant for the current, focal task and initiates the task-related motor responses. The second of these is a ventral system which detects irrelevant stimuli and temporarily interrupts the ongoing processing of the focal task controlled by the dorsal system. This interruption causes temporary motor inhibition to redirect attention towards the unexpected, deviant stimuli. Previous research has shown that the motor inhibition caused by deviants is seen as increased activity in brain areas (i.e., the fronto-basal ganglia neural network; for a discussion, see Wessel & Aron, 2017) and EEG frequency bands (e.g., delta and theta; Wessel & Aron, 2013), considered to be associated with motor inhibition (e.g., Yamanaka & Yamamoto, 2010). Furthermore, the motor inhibition caused by deviant sounds is also seen as a reduction in corticospinal excitability reflected by a decrease in motor-evoked potentials around 150 ms after deviant sound presentation in TMS signals (Wessel & Aron, 2013; see also Dutra et al., 2018). Similar motor inhibition has also been seen in the context of oculomotor activity, whereby the number of eye movements (e.g., saccades and fixations) have been shown to be affected by deviant sounds around 150 ms after sound onset (Graupner et al., 2007). Thus, taken together, these findings support the suggestion that deviant sounds have an inhibitory effect on motor planning, including oculomotor activity.

In the context of reading, oculomotor activity refers to the series of eye movements made to read the text. A large body of research measuring eye movements has shown that readers make a number of fixations (i.e., periods of time when the eyes remain relatively still to maintain central foveal vision) and saccades (i.e., rapid ballistic eye movements designed to move foveal vision from one point to another) when they read. During fixations, several cognitive processes take place that allow readers to visually encode and identify each printed word, integrate them within the sentential context, and ultimately comprehend the text (Rayner et al., 2009). It is these cognitive processes that determine which word is fixated and modulate how long readers fixate each word for. For example, research has shown that readers make more and longer fixations when a word is more difficult to process, which might occur when that word is lower in frequency (e.g., Inhoff & Rayner, 1986), less predictable (e.g., Ehrlich & Rayner, 1981), or longer (e.g., Rayner, 1998).

The tight link that exists between eye movements and the ongoing moment-by-moment cognitive processes as each word is fixated (for a discussion, see Liversedge & Findlay, 2000) has allowed researchers to establish the amount and type of information that is processed during an average eye fixation of approximately 250 ms. That is, readers typically process the word they are currently fixating in foveal vision (i.e., the central 2° of vision) as well as, to some extent, the word(s) in the parafovea (i.e., between 2° and 5° of vision).

Parafoveal processing is typically investigated using gaze-contingent paradigms (e.g., *moving window;* McConkie & Rayner, 1975, and *boundary paradigm*; Rayner, 1975). With these techniques, the stimuli that are displayed can be changed based on where the participant’s gaze is. In experiments with the moving window paradigm (McConkie & Rayner, 1975), readers are presented with a window of text around the position they are currently fixating, while the text around this window is replaced with a mask, usually comprised of x’s. The location of the window changes contingent to readers moving their eyes. Reading performance at smaller window sizes is compared with reading in which no window is used, and performance is examined through computation of several measures including words per minute metric, average fixation duration and average saccade length (e.g., Bélanger et al., 2012). Studies using this paradigm have established that in alphabetic languages like English, 3–4 characters to the left of a fixation and 14–15 characters to the right must be visually available for normal reading to occur (e.g., McConkie & Rayner, 1975). This portion of text is referred to as the *perceptual span* (for reviews, see Rayner, 1998, 2009). Within the body of research exploring the impact of different window sizes, it has been shown that reading performance is significantly poorer when very small window sizes are used to reduce the perceptual span, such that reading cannot proceed normally (e.g., when between two and five characters are visible to the right of fixation; Bélanger et al., 2012; Choi et al., 2015; Leung et al., 2014; Rayner et al., 1981; Veldre & Andrews, 2014; Whitford et al., 2013). Furthermore, in English, research has shown that readers can only acquire information from 12 to 15 characters to the right of fixation (McConkie & Rayner, 1975), with reading performance beginning to asymptote from around 10 characters to the right of fixation (e.g., Bélanger et al., 2012; McConkie & Rayner, 1975). In addition, some studies have shown that when the window size is restricted such that the word in the parafovea is replaced by a mask, reading of the currently fixated word is also affected (e.g., Bélanger et al., 2012; Choi et al., 2015; Veldre & Andrews, 2014). The effect that the characteristics of parafoveal words have on the processing of the currently fixated word is known as *parafoveal-on-foveal effects* (PoF effects; e.g., Kennedy, 1998, 2000, 2008). There exists evidence for both visual and orthographic PoF effects (e.g., Drieghe et al., 2008; Inhoff et al., 2000; Kennedy, 1998; Kennedy & Pynte, 2005; Pynte et al., 2004; Underwood et al., 2000; White, 2008), while limited evidence is available for semantic PoF effects (corpus analysis studies such as Kennedy & Pynte, 2005; Pynte & Kennedy, 2006; Schad et al., 2010; cf. experimental studies such as Brothers et al., 2017; Rayner et al., 1998; White, 2008).

The type of information that is extracted and processed from the parafovea has been the focus of attention in studies using the *boundary paradigm* (Rayner, 1975). When using this paradigm, an invisible boundary is placed before each of the target words embedded in a sentence, and a preview stimulus is displayed in the parafovea. When the reader’s eyes cross the boundary, the preview is replaced by a target word. The characteristics that preview and target stimuli share are then examined to determine the nature of parafoveal processing. Research has established that having a parafoveal preview that shares at least low-level linguistic characteristics (e.g., orthographic and phonological; Chace et al., 2005; Pollatsek et al., 1992) with the target word facilitates the processing of those targets when they are fixated (*preview effects*; Rayner & Pollatsek, 1989). However, the existing models of reading differ on the nature of information that can be processed from the parafovea, along with the mechanisms by which words are lexically processed (for a discussion, see Zang, 2019).

Serial models (e.g., E-Z Reader model; Reichle et al., 1998) suggest that only the currently fixated word is lexically processed at any one time (although see word skipping; e.g., Drieghe et al., 2005; Reichle & Drieghe, 2013), and parafoveal processing begins with a covert shift of attention towards the upcoming word *N* + 1, only after the currently fixated word *N* has been lexically processed. In contrast, parallel models (such as SWIFT; e.g., Engbert et al., 2002, and OB1; e.g., Snell et al., 2018) argue that multiple words are activated and lexically processed within the attentional gradient (or window), and therefore parallel lexical processing occurs for all words within the perceptual span (i.e., both the currently fixated word and some of the words in the parafovea). As a consequence, while there is consensus among models of reading for low-level, visual and orthographic preview effects (for a review, see Schotter et al., 2012), the models disagree on the number of words and the higher-level information that can be processed from the parafovea.

The E-Z Reader Model suggests that orthographic preview effects can be obtained from word *N* + 1 (e.g., Drieghe et al., 2005; White et al., 2008, and for a review, see Rayner, 2009), and from word *N* + 2 when *N* + 1 is skipped (Angele & Rayner, 2011). However, extraction of semantic information is not expected, and high-level linguistic characteristics of parafoveal words should not affect the processing of the currently fixated word (i.e., PoF effects; for a review, see Rayner et al., 2014). However, the SWIFT and OB1 Models suggest that both low-level and high-level (e.g., semantic) preview effects should be seen for word *N* + 1, and to some extent word *N* + 2 (e.g., Snell et al., 2017, 2018) Furthermore, the SWIFT Model accounts also for lexical PoF effects (e.g., Kliegl et al., 2006; Schad et al., 2010).

Yet, the debate that exists between serial and parallel models has recently been challenged, and consequently new hypotheses and models have been put forward. These new accounts suggest that readers might adopt a more flexible approach when reading and treat multi-constituent words (e.g., teddy bear) as a single unit (Multi-Constituent Unit [MCU] hypothesis; for example, Cutter et al., 2014; Yu et al., 2016; Zang, 2019; Zang et al., 2021) or process multiple characters in the perceptual span at the same time to then sequentially activate single words (Chinese Model of Reading; Li & Pollatsek, 2020). According to the MCU hypothesis, when *N* + 1 and *N* + 2 make up a multi-constituent unit and a valid preview of *N* + 1 is displayed, orthographic preview effects on *N* + 2 can be seen, as both words are represented as a single lexical entry (e.g., Cui et al., 2022; Cutter et al., 2014; Yu et al., 2016; Zang et al., 2021). Similarly, the Chinese Reading Model predicts that preview effects can be expected from both characters *N* + 1 and *N* + 2 due to the assumption that characters in the perceptual span are processed in parallel (as seen in Yang et al., 2009). However, in its current form, the Chinese Reading Model cannot make specific predictions about the nature of these effects. That is, the model cannot determine whether these effects extend to semantic preview effects, or whether these are just phonological in nature. In addition, the model currently provides no suggestion of whether PoF effects should be seen (although research into Chinese reading has shown evidence of such effects; e.g., Yang et al., 2009). Despite the differences between models on the nature and location of information extracted from the parafovea, all models agree that once a word is identified, readers programme a saccade towards upcoming words in the sentence, and that this occurs simultaneously to parafoveal processing. That is, whilst programming a saccade towards an upcoming part of the text, readers begin preprocessing this text before their eyes move towards it.

Recent research has suggested that it is the saccadic programming stages of reading that are affected by the presence of deviant sounds. Vasilev and colleagues (2019) used the auditory boundary paradigm (Eiter & Inhoff, 2010) to present deviant (one “deviant” sound within a repeated, steady-state sequence) and standard (repeated, steady-state sequence) sounds while participants read single sentences. By using this paradigm, the researchers manipulated the precise point at which sounds were presented, contingent to the reader’s eye movements. An invisible boundary was placed before each target word embedded in the sentence, and a sound was triggered once the reader’s eyes crossed the boundary. The study showed that deviant sounds caused significantly longer fixation durations on target words than both standard and silent conditions. As saccadic programming is a motor process, and delays in these processes would be reflected as longer fixation durations, the authors concluded that these findings support the notion that deviant stimuli lead to motor inhibition. In addition, the survival analysis (Reingold & Sheridan, 2014, 2018) that Vasilev and colleagues conducted on first fixation duration data for target words revealed that the earliest point at which the deviant and standard sound conditions began to significantly differ was approximately 180 ms after sound onset. The timing of this effect appears similar to the timing shown in previous research on motor inhibition by deviant sounds in picture viewing and verbal reaction time tasks (Graupner et al., 2007; Wessel & Aron, 2013). Thus, this study appears to support the idea that deviant sounds cause motor inhibition, which in the context of reading might be saccadic planning.

Similar results were observed in a following experiment by Vasilev and colleagues (2021). Using the same auditory boundary paradigm, novel deviants (i.e., deviant sounds that change upon each presentation and are therefore “new” to the reader) and standard sounds were presented either at fixation onset or 120 ms after fixation onset on the target words. The timing of the sound presentation allowed for a direct comparison of sounds presented in either the first or second half of a fixation, the latter being considered the period when saccadic programming towards the same (e.g., refixation) or following word takes place, and thus, more saccades are subject to inhibition. The results showed that disruption to reading (as evidenced by longer fixation durations) was significantly higher for deviant sounds compared with standard, and the magnitude of such effects was significantly larger when sound presentation was delayed compared with when presented at fixation onset. Furthermore, using survival analysis (Reingold & Sheridan, 2014, 2018) the authors showed that the effect of deviant sounds appeared 150 ms after sound onset when sounds were presented at fixation, without a delay (supporting the findings of Wessel & Aron, 2013, and Graupner et al., 2007). However, for the 120-ms delay condition, the effect of deviant sounds was shown to appear sooner, only 60 ms after sound onset (i.e., 180 ms after fixation onset). Therefore, taken together, both studies suggest that disruption by deviant sounds is observable after lexical processing is said to occur (e.g., Reichle & Reingold, 2013), and could be attributed to delayed saccadic programming, due to a temporary disruption to oculomotor activity.

While the available literature suggests that poorer reading performance in the presence of deviant sounds may be attributable to saccadic programming inhibition, existing evidence has focussed on the effects of such sounds on foveal processing. However, since models of reading agree that parafoveal processing of upcoming words occurs simultaneous to saccadic programming, it may be possible that deviant sounds also affect parafoveal processing, an aspect of reading which remains unexplored in the context of the auditory deviant effect.

## Current research

**Figure 1.**
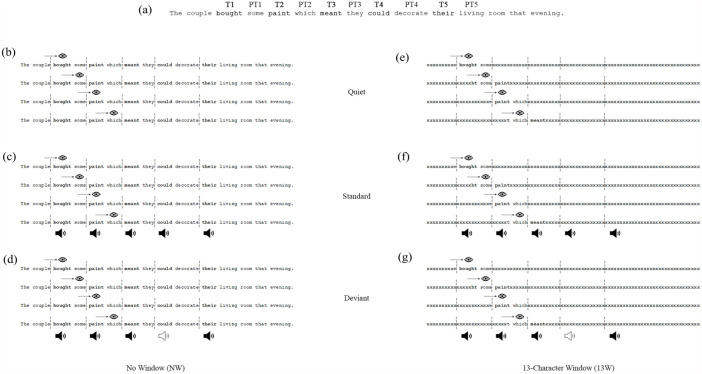
A diagram illustrating the experimental design adopted in the present research. (a) An example sentence indicating the positions of the target words 1, 2, 3, 4, and 5 (i.e., T1, T2, T3, T4, T5), and post-target words 1, 2, 3, 4, and 5 (i.e., PT1, PT2, PT3, PT4, PT5). (b) An example sentence in the No Window-Quiet condition: the whole sentence is visible, and no sound is presented. (c) An example sentence in the No Window-Standard condition: the whole sentence is visible and the same tone is presented on each target word. (d) An example sentence in the No Window-Deviant condition: the whole sentence is visible and the same tone is presented on target words 1, 2, 3, and 5 with a “deviant” tone presented on the remaining (fourth) target word. (e) An example sentence in the 13-Character Window-Quiet condition: the sentence is masked by x’s with a 13-character window of text visible and no sound is presented. (f) An example sentence in the 13-Character Window-Standard condition: the sentence is masked by x’s with a 13-character window of text visible and the same tone is presented on each target word. (g) An example sentence in the 13-Character Window-Deviant condition: the sentence is masked by x’s with a 13-character window of text visible and the same tone is presented on target words 1, 2, 3, and 5 with a “deviant” tone presented on the remaining (fourth) target word.

The aim of this study is to investigate whether auditory stimuli affect foveal and, in particular, parafoveal processing during reading. Readers will be presented with single sentences displayed on the screen according to the auditory boundary paradigm (Eiter & Inhoff, 2010). Standard and deviant sounds will be played contingent to readers fixating five target words embedded in one-line sentences (see Figure 1). The sounds will be 120 ms in length and will be presented 120 ms after fixation onset on each target, to ensure sound presentation will coincide with the second half of an average fixation duration, when saccadic programming and parafoveal processing are thought to occur.

Furthermore, we will adopt the moving window paradigm (McConkie & Rayner, 1975) to manipulate the extent to which readers can extract information from the parafovea. We will use two window sizes (see Figure 1): a 13-character window, with the point of fixation, four characters to the left and eight characters to the right being available on the screen, and no-window, where the entire sentence will be presented. By restricting the perceptual span, while ensuring the currently fixated word *N* is visible to the participants, the 13-character window will allow us to determine whether parafoveal processing is affected by the presence of sound, and whether the nature of sound modulates this effect. This manipulation will also provide an opportunity to corroborate previous well-established results, such that when the portion of visible text is reduced, reading performance will be poorer compared with when the full sentence is presented (e.g., Bélanger et al., 2012). Since research has shown that reading cannot proceed normally at very small window sizes (Bélanger et al., 2012; Choi et al., 2015; Leung et al., 2014; Rayner et al., 1981; Veldre & Andrews, 2014; Whitford et al., 2013) and that reading performance shows little improvement beyond around 10 characters to the right of fixation (e.g., Bélanger et al., 2012; McConkie & Rayner, 1975), we have chosen to use a 13-character window comprising of eight characters to the right of fixation. By using a window of this size, we can ensure that while we can expect to see a reduction in reading performance (since we are reducing the participants’ view to less than 10 characters), the impact of the window should not impair reading too significantly (as would be the case for very small window sizes).

Based on the existing literature on auditory distraction in reading (Vasilev et al., 2019, 2021), we anticipate that in the quiet and standard conditions reading will proceed normally, and readers will be able to initiate parafoveal processing of the upcoming word. Thus, we predict that the nature of the auditory stimulus will modulate both foveal processing of the target words and parafoveal processing of the post-target words. At the target word, we expect to observe comparable reading performance for the quiet and standard conditions, but poorer reading performance for the deviant condition. Similarly, at the post-target word, we expect that reading performance should be comparable for the quiet and standard conditions but poorer for the deviant condition, as in the presence of a deviant sound processing of the post-target word may not be initiated in the parafovea and would need to start upon fixation. Such findings would provide support for the Duplex (e.g., Hughes, 2014; Hughes et al., 2005, 2007) and Unitary (e.g., Bell et al., 2010, 2012; Cowan, 2001) accounts, and further evidence for the hypothesis that deviants cause some form of motor inhibition (e.g., saccadic planning; Vasilev et al., 2019, 2021). Alternatively, if we observe disruption by standard sounds compared with quiet, and greater disruption by deviant sounds compared with standards, these findings would be consistent with the graded attentional account and would suggest that both standard and deviant sounds cause a call for attention, but attentional capture would only occur for deviant sounds (e.g., Bell et al., 2019; Röer et al., 2014a; Schröger et al., 2000).

In line with previous research (e.g., Bélanger et al., 2012; McConkie & Rayner, 1975; Rayner et al., 2010), we also expect to observe significant effects associated with our window sizes. In the 13-window condition, perceptual span is reduced, and therefore we expect poorer reading performance, as parafoveal processing cannot proceed normally. In addition, in the 13-window condition, readers are presented with a string of x’s in the parafovea that is visually dissimilar and does not share any orthographic information with any real word. It is likely that participants might be aware of this unusual non-word-like parafoveal string, and that this might affect reading of the currently fixated word (Angele et al., 2016; Slattery et al., 2011). In contrast, in the no-window condition, readers are able to parafoveally process the upcoming word and extract useful information to be used for later processing, thus maintaining normal reading. Therefore, we expect that at the target word, reading might be poorer in the 13-window, but not in the no-window condition, showing a visual PoF effect (e.g., Kennedy, 1998, 2000, 2008). Similarly, at the post-target word, we predict poorer reading performance in the 13-window condition, as parafoveal preview of the post-target word was not available, but facilitation in the no-window condition, as parafoveal preprocessing of this word could be initiated before being fixated (i.e., showing preview effects; Rayner & Pollatsek, 1989). Although these results cannot distinguish between models of reading (e.g., E-Z Reader, Reichle et al., 1998; OB1, Snell et al., 2018; SWIFT, Engbert et al., 2002; Chinese Model of Reading, Li & Pollatsek, 2020), they will be able to confirm that an appropriate perceptual span is necessary for reading to proceed normally, and that parafoveal processing is a benchmark of efficient reading (Tiffin-Richards & Schroeder, 2015).

Finally, and most critical to the present research, we will test the hypothesis that the nature of the presented sounds modulates parafoveal processing as reflected by a modulation in the effect of window size. We suggest that attentional capture by deviants causes a reduction in attentional focus on the focal task which in turn not only leads to a temporary inhibition of saccadic planning (Vasilev et al., 2019, 2021), but also reduces the perceptual span and the extent of parafoveal processing (thought to occur simultaneously to saccadic planning). Thus, we expect that in the presence of a deviant sound, at the target and post-target words, having a reduced visible portion of text will lead to comparable reading performance as to when the full sentence is presented, since in both cases parafoveal processing will be limited. In contrast, at both target and post-target words, we predict to observe significant differences between 13-window and no-window for the quiet and standard conditions. Readers will be able to initiate parafoveal processing of the upcoming word and to use that information upon fixation on the post-target word in the no-window but not in the 13-window condition. These results would provide evidence for the hypothesis that attentional capture by deviants causes disruption to parafoveal processing. Alternatively, a lack of interaction between sound and window size would suggest that the motor inhibition caused by deviants might be short enough to affect saccadic programming but not the initiation of parafoveal processing.^3^

## Method

### Participants

All participants will be native English speakers, aged between 18 and 30 years and recruited from the University community. Each participant will report normal hearing and normal or corrected-to-normal vision, no learning difficulties associated with reading (e.g., dyslexia) and no neurological disorders (e.g., epilepsy). Participants who are Psychology students at the University of Central Lancashire (UCLan) will be offered course credits, while all other participants will be offered a £15 Amazon voucher as compensation for their time. Ethical approval has been obtained from UCLan’s ethics committee following British Psychological Society ethical guidelines (approval number: SCIENCE 0027). Participants will be asked to provide written informed consent before participating.

### Materials

#### Auditory stimuli

We will use two types of sounds adapted from Vasilev et al. (2021; see https://osf.io/jbsuy for all experimental materials that will be used within this study). The first sound will be a *standard* 400 Hz sine wave tone. The second type of sound, the novel *deviant*, will be the first 42 meaningless environmental sounds (e.g., telephone ringing, engine, etc.) taken and adapted from Vasilev et al. (2021). Each environmental sound will be presented twice throughout the experiment (i.e., twice in the gaze-contingent sound block, but not in the quiet block), as is commonly done within the novel deviant literature (e.g., Escera et al., 1998). All sounds will be monoaural, with a sampling rate of 44100 Hz, a bit depth of 16 bit, and amplitudes will be normalised to be of equal root mean square (i.e., RMS value of –15 dB). Each sound file has been adapted using Audacity 2.3.3 (Audacity Team, 2019), such that they will be 240 ms long, incorporating a 120-ms delay at the beginning before the sound starts playing for 120 ms, which will include a 10-ms fade-in and a 10-ms fade-out.

#### Visual stimuli

The visual stimuli will consist of 252 English sentences, taken from a pool of 300 normed sentences (we will indicate the final 252 sentences within the list of 300 sentences once normed), which are neutral in content. Within the pool of 300 sentences, 62 have been adapted from the stimuli used in Vasilev et al. (2019), and the remaining 238 sentences have been specifically developed for this study. The sentences will be normed before use via an online-based sample of 62 adults, aged 18–30 and recruited from the online platform Prolific Academic (https://www.prolific.com). All participants in the norming study should report normal or corrected-to-normal vision, be native British English speakers and should report no learning difficulties associated with reading. Those who participate in the norming study will receive a payment of £3.90 per half an hour in accordance with the average National Minimum Wage and will not be participants in this study.

Participants will be asked to complete one of two questionnaires which will test either the plausibility and naturalness, or the predictability, of the sentence stimuli. Each questionnaire will contain 300 experimental sentences, and 10 filler sentences designed to ensure participants are completing the questionnaires appropriately. Twelve participants will complete the naturalness and plausibility questionnaire. This group of participants will be asked to read each sentence and rate how plausible and natural it is on a 5-point Likert-type scale (1 = *very implausible/unnatural*, 5 = *very plausible/natural*). Participants will be told that very implausible sentences are those that seem unlikely to be true or valid, and very unnatural sentences are those that appear very unusual to them and are not at all typical for normal English language use, with the opposite being true for very plausible and very natural sentences. Fifty participants will complete the predictability questionnaire. This group of participants will be presented with the contextual frame up to the word before the target and asked to complete each sentence by writing down the first word that comes into their mind. Each participant will see each contextual frame once. Thus, to calculate a predictability score for every target word of each sentence, there will be five versions of the predictability questionnaire. Each version of this questionnaire will include the contextual frames up to a different target word within the sentence (i.e., being the target word 1, 2, 3, 4, or 5) and will be administered to a group of ten participants. We expect that all of the experimental sentences will be rated as highly plausible and natural, and not very predictable.

Sentence stimuli that have been developed for the norming study contain an average of 15.74 words (i.e., 102.21 characters), with a range of 15–19 words (i.e., 84–110 characters). There are five target words (on which sounds will be played) in each sentence which are always presented in word positions 3, 5, 7, 9, and 11 (see Figure 1). After the last target word, sentences contain between four and eight words. Target words are on average 6.2 characters in length (*SD* = 0.99 characters, range = 5–9 characters) and have an average Zipf lexical frequency of 4.69 (*SD* = 0.82, range = 1.81–6.51) as calculated using the SUBTLEX-UK database (Van Heuven et al., 2014). Post-target words are on average 5.46 characters in length (range = 2–13). We will include a table containing the characteristics of the final sentences, as well as target and post-target words, after the norming study is complete.

Sentence stimuli will not contain short function words within the target word region (between words 3 and 11) for several reasons. First, research has shown that short function words are more likely to be skipped (Rayner & McConkie, 1976). Thus, not including this type of word in our sentences will maximise the probability of participants fixating the target and post-target words. Second, by increasing the chance that participants will fixate on every word within this region, sound sequences are more likely to be presented with more regular interstimulus intervals (ISI). Research has shown that continuously changing ISIs do not affect auditory distraction effects (Parmentier & Beaman, 2015). This explains why we do not expect to see an influence of the natural variation in ISIs between sounds presented on target words. Nonetheless, there is evidence to suggest that deviant ISIs may modulate such effects (Hughes et al., 2005), and a deviant ISI might be seen when target word skipping occurs since this would result in a fixation on the following post-target word, which would significantly increase the interval between sounds. Therefore, by increasing the probability of fixation on each target word, we maximise the chances that the observed distraction effects are attributed to the deviant sound stimulus, rather than a deviant ISI produced by word skipping.

### Apparatus

An EyeLink 1000 Plus Desktop Mount will be used to record the participants’ eye movements at a sampling frequency of 2,000 Hz. Participants’ viewing will be binocular, but data will be recorded from one eye only. A chin and forehead rest will be used to stabilise the head and thus avoid head movements being misconstrued as eye movements. The experiment will be presented on a 24.5-inch BenQ ZOWIE XL2540 LCD Monitor and the screen resolution will be set to 1920 × 1080 pixels with a refresh rate of 240 Hz. The experiment will be programmed and run using Experiment Builder (SR Research) on a Gigabyte Ultra Compact PC running Windows 10 Pro.

Visual stimuli will be displayed on a single, left-aligned line in the middle of the screen, in black monospaced Courier New font at 18 pt size on a white background, with each character occupying 15 pixels. The monitor will be positioned 70 cm away from the participants’ eyes and 1° of visual angle will correspond to approximately 2.86 characters. Sentence stimuli will be presented using the moving window paradigm (McConkie & Rayner, 1975). In half of the trials, the sentences will be presented in full (no-window condition; see Figure 1b to d). In the remaining half of trials, a window size of 13 characters will be displayed contingent to the where the participant moves their eyes over the sentence (13-window condition; see Figure 1e to g). In this 13-window condition, the predefined window of 13 characters will be displayed as normal text, while the characters outside this window, including punctuation and spaces, will be replaced with x’s.

Auditory stimuli will be played at 65 dB(A) through Bose QuietComfort 25 noise-cancelling headphones. We will use a UR22mkII Steinberg ASIO soundcard to allow for precise auditory timing and presentation. Auditory stimuli will be presented using the auditory boundary paradigm (Eiter & Inhoff, 2010; Vasilev et al., 2019, 2021; see Figure 1c, d, f and g), whereby an invisible boundary will be placed before each of the five target words. The experiment will contain two blocks, a quiet block of trials whereby no sound will be presented (see Figure 1b and e), and a gaze-contingent sound block which will contain both standard and deviant trials. In the standard trials, all sounds presented will be the same and will appear on all five target words (see Figure 1c and f), and standard sounds will be played on every fixation on target words regardless of whether this is in first- or second-pass reading. By presenting sounds in both first- and second-pass reading, we aim to maintain the regularity of sound presentation (and thus have more regular ISI’s between sounds, thereby minimising the likelihood of temporally deviant ISIs occurring). In the deviant trials, all but one target word will receive the same standard sound in both first- and second-pass reading, with the remaining target word receiving the novel deviant sound in first-pass reading only (see Figure 1d and g). Furthermore, upon refixating the target word within deviant trials (during second-pass reading), if that target word has previously received a “deviant” sound, it will subsequently receive a “standard” sound so as to maintain the auditory deviant effect by presenting only one deviant sound per deviant trial. If instead the word which should receive the deviant sound is initially skipped, but subsequently fixated, it will receive the deviant upon first fixation. Deviant sounds will be presented an equal number of times across trials on either target word 2, 3, or 4. This will ensure that the deviant sound will follow at least one standard sound, and the mental representation of the standard will be re-established by the end of the trial. The blocks will be counterbalanced between participants, whereby half of the participants will complete the quiet block first followed by the sound block, and the remaining participants will complete the sound block first followed by the quiet block.

### Procedure

Participants will be instructed to read the sentence stimuli presented on the screen while ignoring any sounds that may be presented through the headphones. They will then be asked to rest their head and chin on the rests provided before beginning the experiment. Next, we will begin the three-point calibration procedure, during which they will be asked to fixate on each of three dots along a horizontal array. During the experiment, a drift check will be presented before each trial (consisting of a dot appearing on the central left point of the screen) and recalibration will be completed after each block and break, and whenever necessary. The calibration error will be kept at < 0.3° across the experiment to maintain accuracy when contingently presenting the visual and auditory stimuli. All calibration and drift check beeps have been removed from the experiment to maximise the effects of the standard and deviant sounds. This will ensure the participants will only hear the experimental sounds (rather than an additional sound for calibration) which will allow them to identify the standard and deviant sounds clearly. Following the drift check, each trial will begin with a fixation cross (i.e., “+”) on the left side of the screen. Participants will be required to fixate the cross for 500 ms, after which the cross will be replaced by the first letter of the sentence.

It is estimated that the experiment will last for 1 hr 30 min, and will contain 12 practice trials before the formal experimental stimuli are presented. Experimental stimuli will consist of two blocks of trials, counterbalanced between participants. The quiet block will contain 84 trials completed in quiet, with 42 trials presented in the 13-Character Window-Quiet condition and 42 trials presented in the No Window-Quiet condition. The gaze-contingent sound block will contain a total of 168 trials made up of a combination of each sound condition (standard and deviant) within each moving window condition (13-window and no-window), with 42 trials in each condition (13-Character Window-Standard, 13-Character Window-Deviant, No Window-Standard, No Window-Deviant). Trial order will be randomised within each block and counterbalanced by sound condition, moving window condition and sentence. Thus, each participant will receive a different order of sentences in different sound and window conditions, and participants will see each sentence only once.

The participant will silently read the sentence and then press a button on a button box to move to the next trial. Yes/no comprehension questions will be presented after four of the practice trials, and 81 of the experimental sentences (i.e., approximately 32% of the overall trials) to assess comprehension accuracy, and will require a button press response to then begin the next trial. The participants will be under no time constraints when completing the experiment, allowing them to self-pace their reading on a trial-by-trial basis, and to take a break after every 42 trials and whenever they wish.

Upon completion of the experiment, participants will be provided with a questionnaire exploring display change awareness, which will ask them to report if they noticed anything unusual on the display when they were reading the sentences. We anticipate that participants will report that they were aware of the moving window, since previous research has shown that display change awareness is increased when previews within the parafovea are unusual, non-word-like stimuli (i.e., a string of x’s; e.g., Angele et al., 2016; Slattery et al., 2011).

### Power analysis

Power analysis was completed using the PANGEA method described by Westfall (2015; https://jakewestfall.shinyapps.io/pangea/) and based on the results obtained by Vasilev et al. (2021), who adopted the most similar experimental design to this study. We calculated the effect size (Cohen’s *d_z_*) of the distraction effect (i.e., the difference between novel deviant and standard sounds) by using the *t* values that Vasilev et al.’s reported for the first fixation duration (FFD) associated with the distraction effect in the conditions with a 120-ms presentation delay, which is the most similar condition used in this study. This calculation yielded a large effect size of .87. To obtain a power of 0.9 with this effect size and 42 stimuli per condition, a total number of six participants would be required. However, since the interactive effects we aim to examine might be smaller in size, we ran the power analysis based on the average effect size of .3 typically reported in the psychological literature (Brysbaert & Stevens, 2018). Using this effect size and 42 stimuli per condition, we estimated that 72 participants would be necessary to obtain sufficient power of 0.9.

### Proposed data analyses

Before starting data analysis, preprocessing of the data will be conducted by excluding (1) any participants with a comprehension accuracy lower than 80%, (2) trials with first fixation durations shorter than 80 ms or longer than 800 ms using the “clean” function in DataViewer (SR Research), (3) trials on which the sound is presented too early (i.e., before crossing the invisible boundary) or too late (after fixation onset), (4) observations with blinks occurring on the target or post-target words, and (5) deviant trials where the first target word(s) receiving standard sounds are skipped meaning the first sound that is played is a deviant sound. We will remove these trials since readers will hear the deviant sound followed by the standard, and thus may assume the standard is subsequently a “deviant” as it differs from the previous sound.

Eye movement data will be analysed using Generalised Linear Mixed Effects Models (GLMM) in R (version 4.0.5; R Core Team, 2021). Analyses will be completed on raw data, and we will use the “glmer” function within the lme4 package (v.1.1-23; Bates et al., 2015) with binomial family (for skipping), gamma family (for all other local and global measures) and the identity link, to avoid the need for transforming the data (Lo & Andrews, 2015). We intend to use a full random structure as per Barr et al. (2013) for all our models, with sound and window as fixed effects, and subjects and items as crossed random effects (Baayen et al., 2008). We will start by including random slopes for each of the fixed effects and random intercepts for each of the random effects. However, whenever a model does not converge, we will trim the random structure of the model until it reaches convergence. We will do this by reducing the random structure for items first, starting with the removal of correlations between factors, followed by the interactions, and then the random slopes. We will then repeat the same procedure with the random structure for subjects if the model still fails to reach convergence. Successive differences coding contrasts will be used (contr.sdif in the MASS package; Venables & Ripley, 2002, see also Schad et al., 2020) to set up the fixed effect factors. That is, we will run contrasts comparing quiet (level 1) versus standard (level 2), and standard versus deviant (level 3), as well as no-window (level 1) versus 13-window (level 2). The results will be interpreted as significant when the |*t*| or|*z*| value within the GLMM is equal to or greater than 1.96, indicating the results are significant at the .05 alpha level.

Several global and local eye movement measures will be examined in this study to provide insights into the time course of our effects in relation to the entire sentence as well as the target and post-target words respectively. For the global reading measures we will analyse total reading time (the sum of all fixations made on all words within the sentence), average fixation duration (the average duration of all fixations made on all words within the sentence), number of fixations, saccade length, skipping rate (the likelihood of a word not receiving a fixation during first-pass reading), refixation rate (the probability of making another fixation on a word within first-pass reading), probability of regression (the number of regressive saccades made from a later position in the sentence), average first fixation duration (average FFD; the average duration of the first fixation on all words within the sentence), average single fixation duration (average SFD; the average duration of all fixations when only one fixation is made on a word within the sentence), average gaze duration (average GD; the average sum of all consecutive fixations each word within the sentence before making a saccade towards the next word). These measures will provide us with a general view of the nature of reading behaviour that occurs when participants are presented with background sound while restricting their parafoveal preview, as compared with when no sound is presented. We will analyse global reading measures only for the main effect of window for two reasons. First, since we will be unable to discriminate between the effects of standard and deviant sounds (as only one deviant is presented in a string of standards) for both the main effect of sound and interaction between window and sound. Second, because previous research has shown that neither standard nor deviant sounds affect global reading measures (Vasilev et al., 2019). We expect that reading times (total reading time, average fixation duration, average FFD, average SFD, average GD) and some eye movement patterns (number of fixations, probability of regression, refixation rate) will be increased in the 13-window condition compared with no-window. Yet, we predict that the remaining eye movement patterns (skipping rate, saccade length) will be decreased in the 13-window compared no-window condition.

Regarding the local eye movement measures, we will analyse first fixation duration (FFD; the duration of the first fixation on the target or post-target word), single fixation duration (SFD; the duration of the fixation when only one fixation is made on the target or post-target word), gaze duration (GD; the sum of all consecutive fixations on the target or post-target word before making a saccade towards the next word), total viewing time (TVT; the sum of all fixations on the target or post-target word, including those in second-pass reading), and refixation rate (the probability of making another fixation on the target or post-target word within first-pass reading). These measures will provide us with an insight into parafoveal processing that occurs when participants are presented with background sounds while their parafoveal information is restricted, as compared with when no sound is presented. Separate models will be run, one for each eye movement measure, to analyse the main effect of sound, main effect of window and the interaction of sound and window at the target and post-target words for word positions 2, 3, and 4. We expect that for both the target and post-target word, there will be a main effect of sound, whereby reading measures (FFD, SFD, GD, TVT, refixation rate) will be higher for deviant compared with standard and quiet conditions, which will be comparable. Furthermore, we expect there to be a main effect of window whereby reading times and refixation rates on both the target and post-target will be higher for 13-window compared with no-window. While we will examine both of the main effects, the most critical analysis in relation to our hypotheses will be the interaction between sound and window, since our study aims to examine the effect of deviant sounds on foveal and parafoveal processing. We expect there to be a significant interaction between sound and window for the target and post-target word, such that reading times and refixation rates will be higher for 13-window compared with no-window, but only for standard and quiet conditions, and thus, we expect no difference or a smaller difference between window conditions in the presence of deviants.

## Minor deviations from the data collection and analysis plan

Six minor deviations from the pre-registered plan were made. First, eye movement data were analysed using version 4.2.2 of R (R Core Team, 2021) and analyses were completed using v.1.1-32 of the lme4 package (Bates et al., 2015). Second, we note that in our initial manuscript we specified that GLMM’s would be completed using the binomial family for skipping, gamma family for all other local and global measures, and the identity link. However, in order for the GLMM’s to run correctly, we used the binomial family also for global probability of regression, and global and local refixation rates, and whenever we used the binomial family, we used the logit link. Third, we note that the definition of the probability of regression included in our registered experimental design was inaccurate, and therefore the definition we have adopted in the analyses is “the probability of making a regressive fixation on the target or post-target word within second-pass reading.” Fourth, during the preprocessing of the data, we included an additional step whereby we removed any deviant trials where a deviant sound was not played (i.e., when the target word on which the deviant sound was presented was skipped). We included this additional step to ensure that classification of a deviant trial was accurate. These three deviations apply to data preprocessing for both global and local analyses.

Fifth, during data preprocessing for local analyses, we carefully examined the temporal delays of the sound onsets in each trial and noted that sound presentation across the experiment did not occur as we intended. In our registered experimental design, we intended sounds to be played 120 ms after the participants’ gaze crossed the invisible boundary. That is, to be played in the second half of the fixation when saccadic programming and parafoveal processing are thought to occur. To achieve this, sound files incorporated 120 ms of silence at onset, and were programmed to play immediately after the invisible boundary was crossed. However, it became apparent that due to a software error, all trials had a minimum additional delay of 50 ms between crossing the boundary and the onset of the sound file, resulting in a minimum total delay of 170 ms before the sound began playing. Because of this software error, the sound was not played as intended and therefore we had to include one additional step into the preprocessing of the local analyses to test the original hypotheses. To have meaningful results in relation to the effect of sounds on parafoveal processing, we report local analyses which include only observations where the sound was presented in the second half of the fixation and heard for at least 50 ms (as the minimum sound duration shown to produce distraction effects during reading; Vasilev et al., 2019).

Finally, in the pre-registered plan we did not specify how to select the target and post-target words in the standard and quiet conditions for the local analyses. Therefore, during preprocessing of the data, for each sentence in the deviant condition we selected the interest area (i.e., target word) wherein the deviant sound was presented, and then we selected the corresponding interest area for the same sentence in the quiet and standard conditions. This was done for the target word analyses, and the following interest area (i.e., post-target word) was selected for the post-target word analyses. This approach was not specified in the initial registered report, but it was a necessary step to ensure that background sound conditions (quiet, standard, and deviant) were compared for the same target and post-target words appearing in the same sentential positions across conditions. We note that, despite these minor deviations within the preprocessing stages for our global and local analyses, the analyses have been conducted exactly as specified in our pre-registered plan. Additional analyses that we did not specify in our pre-registration document are included in the separate Exploratory Analyses section.

## Results

### Norming study results

A pool of 300 sentences were normed to select the final set of sentences to use in this study. An online-based sample of 62 adults, aged 18–30, were recruited from the platform Prolific Academic (https://www.prolific.com) using the norming study criteria specified in the pre-registered plan. The norming study followed the planned procedure, resulting in the selection of 252 sentence stimuli which contained an average of 15.74 words (i.e., 102.41 characters), with a range of 15–19 words (i.e., 84–110 characters). Target words were on average 6.2 characters in length (*SD* = 0.98 characters, range = 5–9 characters) and had an average Zipf lexical frequency of 4.69 (*SD* = 0.82, range = 1.81–6.51) as calculated using the SUBTLEX-UK database (Van Heuven et al., 2014). Post-target words were on average 5.49 characters in length (range = 2–13).

### Planned global analyses

Thirteen participants were removed prior to data analysis, one due to having a comprehension accuracy lower than 80% (under criterion 1) and 12 due to technical issues during data recording. Therefore, the analyses we report below are based on data from 72 participants (with an average comprehension accuracy of 92%, *SD* *=* 4.29%), in line with the power analysis we conducted. One trial for one participant was lost due to technical issues, resulting in a total of 18143 trials included for preprocessing. 9.6% of fixations were removed by filtering blinks before/after fixation out (under “data filters” within the data preferences) and using the “Perform 4-Stage Fixation Cleaning” function in DataViewer (SR Research). 42.55% of trials were removed because sounds were presented too early or too late in spatial terms, that is on the incorrect interest area (criterion 3), 0.36% of trials were removed due to being a deviant trial with no deviant sound presented (under the additional criteria specified in the Minor Deviations from Data Collection and Analysis Plan section), and 0.01% of trials were removed because the first sound played was a deviant sound (criterion 5). A total of 10356 (57.08%) cleaned trials out of the initial 18144 trials were included in the final dataset for analysis.^4^

We predicted that global reading times (Total Reading Time, TRT; Average Fixation Duration, AvFD; Average First Fixation Duration, AvFFD; Average Single Fixation Duration, AvSFD; Average Gaze Duration, AvGD) and some global eye movement patterns (Number of Fixations, FC; Probability of Regression, Reg Prob; Refixation Rate, Global RR) would be increased in the presence of a window compared with when no window was present. This expectation was based on the findings of previous research showing disruption to reading due to the limited amount of parafoveal information that can be processed under window reading conditions (e.g., Bélanger et al., 2012; Choi et al., 2015; Leung et al., 2014; Rayner et al., 1981; Veldre & Andrews, 2014; Whitford et al., 2013). In addition, for the same reason, we predicted that Global Skipping (Skip) and Saccade Length (SL) would be decreased in the window compared with the no-window condition, as shown in previous studies (e.g., Häikiö et al., 2010; Rayner et al., 2010).

Our global analyses showed that, as predicted, reading times were increased in the window compared with the no-window condition (window effect for TRT: 89 ms, AvFD: 8 ms, AvFFD: 8 ms, AvSFD: 9 ms, AvGD: 12 ms; see Table 1 for descriptive statistics and GLMM statistics for global measures). Furthermore, as expected, global skipping was lower and saccade length was shorter in the window compared with no-window condition, and refixation rate was marginally larger for the window compared with no-window condition. However, contrary to our expectations, the number of fixations and probability of regression were reduced, not increased, in the window compared with no-window condition.

**Table 1.** Mean descriptive statistics (SDs) and fixed effects estimates for the global measures for the global, sentence-level data.

Global Eye Movement Measures					
Measures	Descriptive Statistics		GLMM (Window vs. No-Window)		
	Window	No-Window	*b*	*SE*	*t*
TRT (in ms)	4,256 (1,944)	4,167 (1,992)	86.22	3.18	**27.14**
AvFD (in ms)	254 (37)	246 (37)	9.04	1.53	**5.91**
AvFFD (in ms)	236 (43)	228 (45)	9.65	1.80	**5.36**
AvSFD (in ms)	236 (44)	227 (44)	10.92	2.14	**5.12**
AvGD (in ms)	295 (80)	283 (82)	13.89	2.94	**4.73**
FC	24 (10)	25 (11)	–1.16	0.36	–**3.23**
SL (in characters)	5.63 (0.72)	6.18 (0.77)	–0.20	0.03	–**6.02**
Skip (%)	2% (5%)	4% (7%)	–0.85	0.08	–**11.26**
Reg Prob (%)	14% (19%)	21% (21%)	–0.72	0.07	–**10.18**
Global RR (%)	25% (20%)	24% (20%)	0.07	0.04	1.74

*Note.* Statistically significant GLMM results are presented in **bold**, and results approaching significance are underlined. TRT = Total Reading Time; AvFD = Average Fixation Duration; AvFFD = Average First Fixation Duration; AvSFD = Average Single Fixation Duration; AvGD = Average Gaze Duration; FC = Number of Fixations; SL = Average Saccade Length; Skip = Skipping Rate; Reg Prob = Probability of Regression; Global RR = Refixation Rate.

### Planned local analyses

A total of 10,356 trials (57.08% of the initial 18,144 trials) that were cleaned for global analyses were further pre-processed to enter in the local analyses. During preprocessing, as specified under the Minor Deviations from Data Collection and Analysis Plan section, we also removed data points associated with of 3.38% (1,658) of interest areas for which there was an additional delay of over 100 ms for the local analyses (see Figure S1 in the data provided in the OSF link for the distribution of delays). Second, 7.58% trials were removed due to the participant skipping the interest area selected for analysis. Finally, 10.63% of trials were removed due to the participant hearing less than 50 ms sound during the first fixation duration (which is the minimum sound duration shown to produce distraction effects during reading; Vasilev et al., 2019). This resulted in the inclusion of a total of 7,053 cleaned trials for local analyses, that is, 49% of trials out of the initial 18,144 trials.

#### Target word analyses

##### Effect of sound

We predicted that, at the target word, there would be a main effect of sound, whereby reading measures (FFD, SFD, GD, TVT, RR) would be higher for deviant compared with standard and quiet conditions, which would be comparable. The analyses on the target word showed that, as expected and in line with previous findings (e.g., Vasilev et al., 2019, 2021), reading times were significantly longer in the deviant condition compared with the standard condition (difference between deviant and standard for FFD: 23 ms, SFD: 22 ms, GD: 37 ms, TVT: 92 ms; see Table 2 for descriptive statistics and Table 3 for GLMM statistics for local measures on the target and post-target words). However, contrary to our expectations, reading times were significantly longer and refixation rates were lower in the standard condition compared with the quiet condition (difference between standard and quiet for FFD: 84 ms, SFD: 81 ms, GD: 74 ms, TVT: 22 ms, RR: –5%).

**Table 2.** Mean descriptive statistics for the local eye movement measures on target and post-target words on which sounds were played (SDs in parentheses).

Local Eye Movement Measures						
*Target Word*						
Measure	Q-NW	Q-W	S-NW	S-W	D-NW	D-W
FFD (in ms)	235 (79)	240 (77)	330 (91)	312 (78)	353 (98)	334 (94)
SFD (in ms)	235 (76)	239 (75)	324 (86)	311 (74)	348 (94)	330 (86)
GD (in ms)	269 (119)	274 (114)	354 (128)	337 (102)	392 (149)	372 (159)
TVT (in ms)	360 (239)	339 (216)	384 (185)	358 (143)	480 (258)	445 (263)
RR (%)	16% (36%)	16% (37%)	10% (30%)	12% (33%)	15% (36%)	14% (34%)
Post-target Word						
Measure	Q-NW	Q-W	S-NW	S-W	D-NW	D-W
FFD (in ms)	241 (87)	242 (78)	256 (90)	270 (103)	267 (97)	268 (95)
SFD (in ms)	241 (86)	239 (74)	260 (89)	271 (103)	263 (91)	262 (87)
GD (in ms)	275 (124)	271 (113)	291 (120)	295 (124)	297 (138)	304 (144)
TVT (in ms)	365 (241)	340 (230)	314 (163)	299 (132)	384 (230)	363 (235)
RR (%)	16% (37%)	13% (34%)	16% (37%)	12% (32%)	13% (34%)	15% (36%)

Note: FFD = First Fixation Duration; SFD = Single Fixation Duration; GD = Gaze Duration; TVT = Total Viewing Time; RR = Refixation Rate.

**Table 3.** Fixed effects estimates for local eye movement measures on the target and post-target words for all window and sound conditions.

Local Eye Movement Measures															
*Target Word*															
	Window vs. No-Window			Quiet vs. Standard			Standard vs. Deviant			Window × Quiet vs. Standard			Window × Standard vs. Deviant		
	*b*	*SE*	*t*	*b*	*SE*	*t*	*b*	*SE*	*t*	*b*	*SE*	*t*	*b*	*SE*	*t*
FFD	–9.86	2.88	**–3.43**	69.48	8.18	**8.49**	23.38	5.37	**4.35**	–23.03	4.38	**–5.26**	1.07	5.30	0.20
SFD	–8.41	2.90	**–2.90**	59.97	5.20	**11.54**	24.59	5.10	**4.82**	–21.77	5.25	**–4.15**	2.19	6.38	0.34
GD	–11.56	3.18	**–3.64**	56.33	3.60	**15.67**	35.80	3.64	**9.83**	–24.40	3.31	**–7.37**	–5.01	4.30	–1.18
TVT	–29.33	4.37	**–6.72**	17.68	4.55	**3.89**	69.85	5.48	**12.74**	–7.41	3.18	**–2.33**	–13.33	4.05	**–3.29**
RR	–0.05	0.14	–0.36	–0.62	0.20	**–3.10**	0.40	0.25	1.63	0.20	0.30	0.65	–0.49	0.35	–1.40
Post-Target Word															
	Window vs. No-Window			Quiet vs. Standard			Standard vs. Deviant			Window × Quiet vs. Standard			Window × Standard vs. Deviant		
	*b*	*SE*	*t*	*b*	*SE*	*t*	*b*	*SE*	*t*	*b*	*SE*	*t*	*b*	*SE*	*t*
FFD	10.09	4.33	**2.33**	–3.65	4.35	–0.84	9.36	6.24	1.50	8.32	4.17	**2.00**	1.10	5.85	0.19
SFD	10.69	4.60	**2.33**	–4.16	6.40	–0.65	6.16	7.13	0.86	14.54	5.64	**2.58**	–1.62	6.74	–0.24
GD	7.38	4.21	1.75	–6.21	4.48	–1.39	7.32	5.89	1.24	3.61	5.36	0.67	15.81	7.22	**2.19**
TVT	–14.16	4.71	**–3.01**	–41.91	4.54	**–9.23**	41.26	4.90	**8.42**	8.67	5.23	1.66	–1.90	6.21	–0.31
RR	–0.14	0.12	–1.13	–0.12	0.16	–0.77	–0.01	0.18	–0.07	–0.27	0.30	–0.90	0.70	0.36	**1.98**

*Note.* Statistically significant results are presented in **bold**, and results approaching significance are underlined.

##### Effect of window

We predicted a main effect of window whereby reading times and refixation rates on the target word would be higher for the 13-window compared with no-window condition. Contrary to our predictions, on average, across all sound conditions (quiet, standard, deviant), reading times were longer for the no-window compared with the window condition (difference between no-window and window condition for FFD: 11 ms, SFD: 9 ms, GD: 11 ms, TVT: 27 ms). However, these main effects were driven by an interactive effect (see below). No significant effect of window was seen for refixation rates.

##### Interactive effect of sound and window

We predicted that for both quiet and standard conditions, reading times and refixation rates would be higher in the window condition compared with no-window condition, with the effect of window being comparable in the two sound conditions. We expected no difference, or a smaller difference, between window conditions in the presence of deviant sounds as compared with a larger difference between window conditions in the presence of standard sounds.

When considering the early reading time measures (e.g., FFD, SFD, and GD), contrary to our expectations, a significant interaction was found when comparing window conditions between quiet and standard sounds, but not between standard and deviant sounds. We observed a smaller difference between window and no-window in the quiet condition as compared with in the standard condition, for which the difference was larger. Simple effect analyses using version 1.10.0 of the emmeans package (Lenth et al., 2024) showed that for the quiet condition, the early reading time measures were shorter for the no-window condition compared with the window condition (significant difference of 4 ms for SFD, *b* = –5.380, *SE* = 1.830, *z* = −2.932, *p* = .0034; marginally significant difference of 5 ms for GD, *b* = –6.380, *SE* = 3.400, *z* = −1.875, *p* < .0001). However, in the presence of standard sounds, a directionally opposite effect was seen, such that reading times were shorter when a window was present as compared with no-window (significant difference of 18 ms for FFD, *b* = 17.890, *SE* = 3.790, *z* = 4.727, *p* < .0001; 13 ms for SFD, *b* = 16.390, *SE* = 5.150, *z* = 3.184, *p* = .0015; 17 ms for GD, *b* = –18.020, *SE* = 3.880, *z* = 4.640, *p* < .0001).

When we consider the later measure of TVT, contrary to our predictions, a significant interaction was found when comparing window conditions between quiet and standard sound conditions. Simple effects analyses showed a smaller difference between window and no-window in the quiet condition (significant difference of 21 ms, *b* = 19.900, *SE* = 4.700, *z* = 4.248, *p* < .0001) as compared with in the standard condition (significant difference of 26 ms, *b* = 27.400, *SE* = 4.590, *z* = 5.958, *p* < .0001), with reading times being significantly longer for the no-window condition compared with the window condition. As expected, we found a significant interaction between standard and deviant sounds, with a larger difference seen between window and no-window for deviant (significant difference of 35 ms, *b* = 40.700, *SE* = 5.670, *z* = 7.172, *p* < .0001) as compared with standard sound conditions. However, contrary to our expectations, reading times were significantly longer in the no-window condition compared with the window condition. Finally, no significant interactive effects of sound and window were seen for refixation rates.

#### Post-target word analyses

##### Effect of sound

At the post-target word, we predicted that there would be a main effect of sound, whereby reading measures (FFD, SFD, GD, TVT, RR) would be higher for deviant compared with standard and quiet conditions, which would be comparable. As expected, we did not find any significant effects in the early reading time measures (FFD, SFD, GD) and refixation rates between quiet and standard conditions. However, contrary to our predictions, no significant effects on early reading time measures and refixation rates were found between standard and deviant conditions. Finally, when considering the later measure of TVT, as predicted, reading times were significantly longer for the deviant condition compared with standard (difference between deviant and standard for TVT: 67 ms). Again, contrary to our expectations, we found a significant difference between standard and quiet conditions, such that reading times were shorter for standard compared with quiet (difference between quiet and standard for TVT: 46 ms).

##### Effect of window

At the post-target word, we expected there to be a main effect of window whereby reading times and refixation rates would be higher for the 13-window compared with no-window condition. As expected, early reading time measures were significantly longer for the window compared with the no-window condition (difference between window and no-window for FFD: 5 ms, SFD: 3 ms, marginal effect for GD: 2 ms). For the later measure of TVT, the effect of window at the post-target word was in the opposite direction (namely, longer TVTs for the no-window compared with window condition), but the direction of this effect was consistent with that observed for all measures at the target word.

##### Interactive effect of sound and window

As with the target word, at the post-target word we predicted that there would be a significant interaction between sound and window, such that for both the quiet and standard conditions, reading times and refixation rates would be higher in the window condition compared with the no-window condition, with the effect of window being comparable in the two sound conditions. We expected no difference, or a smaller difference, between window conditions in the presence of deviant sounds compared with standard sounds.

When considering the early reading time measures, at the post-target word, there was a significant interaction between window and sound conditions. As expected, in the quiet and standard conditions, reading times (FFD, SFD) were significantly longer in the window compared with the no-window condition. However, unexpectedly, the difference was larger for the standard compared with the quiet conditions (difference between window and no-window in the quiet condition for FFD: 1 ms, SFD: 2 ms; difference between window and no-window in the standard condition for FFD: 14 ms, SFD: 11 ms). Simple effects analyses showed that while differences between window conditions were not significant in the quiet condition (FFD, *b* = –4.170, *SE* = 4.000, *z* = –1.044, *p* = .2966; SFD, *b* = –1.540, *SE* = 4.440, *z* = –0.346, *p* = .7291), they were significant in the standard condition (FFD, *b* = –12.490, *SE* = 4.710, *z* = –2.654, *p* = .0079; SFD, *b* = –16.080, *SE* = 6.030, *z* = –2.667, *p* = .0076).

Furthermore, we found a significant interaction between window conditions in the standard and deviant conditions in the slightly later measure of GD, such that reading times were longer in the window compared with the no-window condition. Simple effects analyses showed that this difference was larger and significant for the deviant (significant difference of 7 ms, *b* = –19.123, *SE* = 7.310, *z* = –2.615, *p* = .0089) compared with the smaller and non-significant difference between window conditions for the standard condition (non-significant difference of 4 ms, *b* = –3.315, *SE* = 5.680, *z* = –0.584, *p* = .5592). Furthermore, we saw a significant interaction in refixation rates between standard and deviant conditions. In the standard condition, refixation rates were higher in the no-window compared with window condition, and the opposite direction of this effect was seen in the deviant condition. However, simple effects analyses showed that these differences were not significant (non-significant difference of –4% for the standard condition, *b* = 0.463, *SE* = 0.289, *z* = 1.600, *p* = .1095; non-significant difference of 2% in the deviant condition, *b* = –0.241, *SE* = 0.206, *z* = –1.172, *p* = .2411).

### Exploratory local analyses

To explore the interactive effects of sound and window seen in the total viewing times at the target word, we undertook additional exploratory analyses investigating the probability of a regression to the target word (see Table 4 for descriptive and GLMM statistics for the regression analyses). The analyses revealed a main effect of sound, such that the probability of a regression to the target word was significantly higher in the quiet condition compared with the standard condition (5% difference between quiet and standard for probability of regression), however no significant differences were seen when comparing standard to deviant sound conditions. Furthermore, no significant main effect of window and no interactive effects of sound and window were found.

**Table 4.** Mean descriptive statistics (SDs in parentheses) and fixed effects estimates for the exploratory analyses for the local probability of regression on the target words on which sounds were played.

Descriptive Statistics			
Window Condition	Quiet	Standard	Deviant
No-Window	16% (36%)	10% (30%)	15% (36%)
Window	16% (37%)	12% (33%)	14% (34%)
GLMM Statistics			
	*b*	*SE*	*t*
Window vs. No-Window	–0.05	0.14	–0.36
Quiet vs. Standard	–0.62	0.20	**–3.09**
Standard vs. Deviant	0.40	0.25	1.63
Window × Quiet vs. Standard	0.20	0.30	0.65
Window × Standard vs. Deviant	–0.49	0.35	–1.40

*Note.* Statistically significant results are presented in bold.

## Discussion

The aim of this study was to examine whether background sound influences the extent of the perceptual span during reading by analysing readers’ eye movements during an eye-tracking experiment using the auditory boundary paradigm (Eiter & Inhoff, 2010) in conjunction with the moving window paradigm (McConkie & Rayner, 1975). Previous eye movement studies have shown significant disruption to reading by deviant sounds, which researchers suggested to be attributable to an inhibition of saccadic programming (Vasilev et al., 2019, 2021). If the deviance effects in the existing literature are a result of inhibition of saccadic programming, it is possible that such effects will extend to parafoveal processing given that models of reading agree that parafoveal processing of upcoming words occurs simultaneous to saccadic programming (e.g., E-Z Reader model; Reichle et al., 1998, SWIFT; Engbert et al., 2002, and OB1; Snell et al., 2018).

In line with our predictions and previous findings (e.g., Bélanger et al., 2012; Choi et al., 2015; McConkie & Rayner, 1975; Rayner et al., 2010; Veldre & Andrews, 2014), our global analyses revealed more disruption to reading in the window condition compared with the no-window condition, as indexed by increased reading times, reduced skipping and reduced saccade amplitude in the presence of a window compared with no window. These effects are indicative of disruption to parafoveal processing due to the restricted perceptual span in the presence of a window relative to when no window was present. It is notable that regression probability and fixation count measures were reduced in the window condition compared with the no-window condition. Ordinarily, one might anticipate increased regression probability and number of fixations under reading conditions that are more, rather than less, difficult. Thus, these effects were not anticipated. Given that participants were less likely to make regressive fixations in the presence of a window, it makes sense that the overall fixation count (which includes both first- and second-pass fixations) was reduced. However, it remains interesting that even though readers clearly experienced disruption to reading (as shown by the other global measures), the disruption did not cause them to re-read the sentences. One possibility is that because reading times were longer, and skipping rate and saccade length were reduced, then readers did not need to make regressions, and consequently, overall, more fixations when reading in the window condition. Of course, this suggestion is speculative, and it would mean that readers were trading off regressions and number of fixations in favour of slower reading when a moving window constrained their perceptual span.

When we consider the results of our local analyses, significant effects of sound and window were seen. Starting with the effect of sound at the target word, in line with our expectations, disruption to reading was greater for deviant sounds compared with standard sounds (in line with previous research; e.g., Vasilev et al., 2019, 2021). However, contrary to our expectations, standard sounds were more disruptive to reading than quiet. To some extent, our findings provide support for a graded attentional account of auditory distraction (e.g., Bell et al., 2019; Röer et al., 2014a; Schröger et al., 2000), which would suggest that both standard and deviant sounds produced a call for attention (i.e., a temporary withdrawal of resources away from the reading task to evaluate whether a full attentional switch towards the background sound was needed), but attentional capture (i.e., a full attentional switch away from the reading task) would have only occurred for the deviant sounds. However, our findings may also provide support for the Duplex (e.g., Hughes, 2014; Hughes et al., 2005, 2007) and Unitary (e.g., Bell et al., 2010, 2012; Cowan, 2001) accounts of auditory distraction. Research supporting the Duplex account has explored the impact that changing-state and deviant stimuli have on task performance and found that both changing-state and deviant stimuli produce disruption relative to quiet, but deviant sounds produce disruption over-and-above changing-state sounds (Hughes et al., 2005, 2007). For example, a voice deviant produces disruption of the same magnitude regardless of whether it occurs in the context of a steady-state sequence (e.g., AAAAA) or a changing-state sequence (e.g., ABABA; Hughes et al., 2005, 2007) which suggests that attentional capture does not arise as a result of the presence of changing-state stimuli, and instead it is the presence of a deviant that captures attention. In light of this, it may be possible that the continuously changing intervals occurring between the sounds in this study may have elicited changing-state qualities in both our standard (cf. Jones & Macken, 1995) and deviant conditions, thus yielding disruptive effects in comparison to quiet, with deviant being more disruptive than standard. That is to say, while the deviant sound used in this study was somewhat similar to that typically used in research supporting the Duplex and Unitary accounts, the standard was categorically different, and this may be a possible reason why we saw differences between performance in the presence of standard sounds and silence. However, given the evidence that changing-state sequences do not produce attentional capture (Hughes et al., 2005, 2007) some further work is required to uncover the basis of the effect of the standard sounds on reading.

It is, however, important that we acknowledge that the irregularity with which the sounds were presented would have existed regardless of the software error, since the sound presentation was dependent on the duration for which the participants’ fixated the target and post-target words. To be clear, the total time that the readers’ spent on the target and post-target word before leaving to fixate another word (i.e., the gaze durations), along with the likelihood of fixating the target and/or post-target words, determined the intervals with which the sounds were presented. Given that there is natural variability in a readers’ gaze duration dependent on a number of factors (e.g., word frequency, word length, and word predictability; Clifton et al., 2016), it is reasonable to expect inter-stimulus irregularity even without the additional delays that occurred in this study. Having said this, research has shown temporally irregular stimuli to be less disruptive than temporally regular stimuli (Parmentier & Beaman, 2015). And indeed, previous eye movement studies adopting the same auditory manipulation have shown no difference between reading in silence and in the presence of standard (temporally deviant) sounds, compared with novel (temporally and tonally deviant) sounds, which have been shown to cause significant disruption to reading (Vasilev et al., 2019, 2021). Thus, the present finding that standard sounds cause significant disruption to reading relative to quiet (where no such disruption is seen) does not align with previous studies exploring the effect of background sound during reading. Nevertheless, in the context of serial recall tasks, disruption by standard sounds has been shown relative to quiet (e.g., Bell et al., 2019; see also Parmentier & Beaman, 2015 for effects of standard sounds regardless of presentation irregularity). Thus, it may be reasonable to see similar graded disruption across quiet, standard, and deviant sound conditions in reading tasks too. Further research is needed to examine what factors might determine significant differences when comparing standard, repeated, sounds in relation to reading in silence.

It is important to note that the disruptive effects of standard and deviant sounds are evident throughout all stages of the reading process. To be clear, the disruptive impact of a sound presented during reading was present in the earliest local measures capturing fixations made as soon as a target word was fixated and prior to the eyes leaving the word (FFD, SFD and GD), as well as in the later measure of TVT that indexed reading time differences during initial processing of target words as well as later processing of those words during re-reading. This result is important, in that it shows that auditory distraction appears to influence processes from the earliest stages through to later stages of sentence processing. Ordinarily, effects observed in early first pass measures are associated with word identification (e.g., Inhoff, 1984; Juhasz & Rayner, 2003; though effects can arise due to syntactic and semantic processing difficulties in these measures; Staub, 2010, 2015; Staub et al., 2007), while later measures are often taken to reflect later aspects of processing such as the computation of a representation of sentential meaning, or even discourse integration. If we had observed disruption for earlier but not later reading time measures, this would suggest that early (e.g., word identification) processes, but not later (e.g., sentence integration) processes, were disrupted by the presence of sounds. And of course, if auditory stimuli affected only late, but not early measures, conversely, this would suggest that integration rather than early processes such as word identification were affected. However, the fact that we observed disruption across early and late measures suggests that auditory stimuli were disruptive at a generic level, rather than affecting particular stages of linguistic processing. Furthermore, we note that the auditory stimuli we used in this study were non-linguistic in nature, suggesting that the effects we observed were unlikely to be attributable to interference effects in respect of aspects of linguistic processing. Perhaps these results suggest that the distraction effect produced by the auditory stimuli was dependent on the amount of parafoveally extracted information.

In contrast, when we consider the effects of sound on the post-target word, we see a somewhat different pattern of effects. While significant disruption to the early reading time measures (FFD, SFD, GD) was seen at the target word, no such effects of sound were seen at the post-target word. Furthermore, while TVT at the post-target word was longer in the deviant compared with standard sound conditions (as seen at the target word), TVT was longer in the quiet compared with standard sound conditions (which is the opposite to the effects seen at the target word). When considering these differences, the first point to note is that reading in the quiet condition was very comparable across the target and post-target word. This is not at all surprising given the identical auditory reading conditions at both word positions—there was no sound onset at either word. The second point to note is that in the standard and deviant conditions, at the target word, readers heard a sound stimulus, whereas at the post-target word they (almost always) processed the word in silence (the sound presentation continued briefly during the initial fixation on the post-target word on only 13% of fixations). That is, reading of the target and post-target word occurred under very different auditory conditions (sound vs. no sound). Assuming that the presence of sound will result in more distraction than the absence of sound, it is not surprising that this is exactly what we observed. Thus, it appears that pronounced effects of the auditory stimulus occurred at the word that was fixated when the sound was played, and these effects were greater for deviant than standard sounds. Since we found significant effects of sound on the target but not the post-target word, it may be suggested that effects of the background sounds were short-lived and contained to fixations on the target word during which the sound was presented. However, while the effects at the post-target word in the early reading time measures (FFD, SFD, GD) were not significant, the means show a similar pattern of disruption due to these auditory stimuli (i.e., reading times were longer in the standard condition relative to quiet, and longer in the deviant condition relative to standard). Thus, it may be suggested that the effects of the sound, that is, disruption as indexed by increased fixation durations, spilled over and persisted during processing on the post-target word, though to a much-reduced degree since the sound presentation had (almost always) ended at the point that the post-target word was first fixated. In relation to the TVT, we remind the reader that this measure represents the overall time that readers spent processing that word in the sentence, and thus, it captures rereading as well as initial reading of the word, which itself is likely to reflect the degree to which readers engaged in re-reading of the sentence more generally. We will consider the issue of patterns of re-reading in more detail shortly.

When we consider the effect of window at the target word, at first glance the pattern of results appears more complicated than we had anticipated, and to this extent the explanations we offer are post hoc. Despite the complexity, the pattern of results is very interesting, and we believe, meaningful. Specifically, at the target word, the effect of window was in the opposite direction to that described previously, that is, reading times in this study were longer in the no-window compared with the window condition. However, it is important to note that these effects were driven by the interactive effects of sound and window. That is, when we consider the effect of window in the quiet conditions, which is the typical manipulation in which the moving window paradigm has been used in all previous studies (e.g., Bélanger et al., 2012; McConkie & Rayner, 1975), for early reading time measures we see an effect that is identical in direction to that observed in the previous research; reading times were longer in the window compared with no-window condition. However, when we consider the effect of window in the standard and deviant conditions (i.e., conditions under which window manipulations of reading have not before been examined), an effect in the opposite direction is observed, such that reading times were shorter in the window compared with no-window condition. When considering these effects, it is important not to overlook the fact that when participants were reading sentences without a window, then any effect on reading at the target word must have arisen as a direct consequence of the sound alone. And it was clearly the case that auditory stimuli presented at the target word did produce disruption to reading at that word relative to reading under conditions of quiet. Nonetheless, this leaves us with the less straightforward question of why reading was less disrupted when a sound (whether standard or deviant) was presented in conjunction with a window restricting readers’ viewing, compared with when sounds occurred during reading without a window. A possible explanation for this is that because participants were unable to effectively process upcoming words in the parafovea (due to the presence of a window hindering parafoveal processing of upcoming information), they curtailed processing of the fixated word and made fewer and shorter fixations. Readers might have done this to move their eyes more rapidly to parafoveal locations, because it was only upon fixation of words in the parafovea that useful linguistic information about them became available.

Next, when we consider the effect of window at the post-target word, a typical window effect was seen, such that reading times (FFD, SFD, GD) were longer in the window compared with no-window condition (in line with previous research; e.g., Bélanger et al., 2012; McConkie & Rayner, 1975). This is unsurprising given that, as previously mentioned, participants were reading the post-target word on the vast majority of instances when no sound was played (regardless of sound condition), which is the typical reading condition under which the window effect has previously been observed. However, when we consider the later measure of TVT, we saw a directionally opposite effect of window, such that total viewing times were longer in the absence of a window as compared with when a window was present. It is important to note that the direction of this effect was consistent with that observed for all measures at the target word, and thus, it may be the case that this effect is linked to patterns of re-reading seen at the target word. That is, it may be the case that the presence of a window causes a reduction in the probability and/or duration of regressive fixations in second-pass reading on the post-target word. We will return to the discussion of re-reading fixations when we discuss the TVT effects in more detail below.

We next consider the early reading time measures at the post-target word in relation to the interactive effects of sound and window. Recall that we observed interactive effects of sound and window at this region, but the pattern of effects was different from that observed at the target word. In line with the effects seen at the target word, reading times for the early measures (FFD, SFD, GD) at the post-target word were longer in the presence of a window compared with no window under quiet reading conditions (in line with the effects seen at the target word, and with previous research; e.g., Bélanger et al., 2012; McConkie & Rayner, 1975). As previously mentioned, this is unsurprising as reading conditions at both target and post-target regions in the quiet sound condition were identical, and thus, we would expect comparable reading across the sentence. In contrast, when a sound was present, we see a directionally opposite effect at the post-target word as compared with the target word. Recall, at the target word reading in the presence of a sound (whether standard or deviant), resulted in longer reading times in the no-window condition as compared with the window condition, with comparable effects for standard and deviant sounds. However, at the post-target word, reading times were longer in the window compared with no-window condition, and the measure on which these effects appeared differed dependent on the sound that was presented. That is to say, the disruptive effects across the sound conditions showed a differential time course of effects. Specifically, the difference between window conditions was larger for the standard compared with the quiet condition; that is to say, the presence of a window was more disruptive to the earliest stages (FFD and SFD) of reading when participants were presented with a standard sound compared with when no sound was presented. However, when we consider the effect of a deviant sound compared with a standard sound, while the direction of the effects was the same (i.e., window producing longer reading times than no-window), these effects were only seen on the slightly later measure of GD, such that the difference between window conditions was larger for the deviant compared with the standard condition. These results suggest a slightly extended time course for deviant sounds as compared with standard sounds.

To us, the interactive effects seen at the post-target word raise two important questions. The first concerns why readers spent longer processing the post-target word in the window than the no-window condition in the presence of standard and deviant sounds, when at the target word reading times were longer in the no-window condition. That is, why did the window effect switch direction between the target and the post-target words in the presence of sounds? We suggest that the switch in the direction of the window effect across words in the standard and deviant sound conditions is related to our suggestion that when readers were fixating the target word, they undertook more effective parafoveal processing of upcoming words (including the post-target word) in the no-window than the window condition. Consequently, we suggested that they moved their eyes to the post-target word more rapidly when reading with, than without, a window. If this was the case, then when readers initially fixated the post-target word, under window reading conditions, they would have pre-processed that word to a lesser degree than was the case under no-window reading conditions. Thus, it may have been for this reason that we observed longer reading times at the post-target word under window than no-window reading conditions. While this post hoc explanation is speculative, it does account for the switch in direction of the window effect across target and post-target words.

The second question concerns why the disruption to processing that occurred under the window reading conditions was delayed for deviant compared with standard sounds. Clearly, as we noted above, the general disruption to processing that we observed for reading under window relative to no-window conditions likely arose because readers were less able to effectively process the post-target word in the parafovea. However, across the FFD, SFD, and GD measures it was the case that for the earlier measures (FFD and SFD) both the standard and the deviant sounds produced disruption to processing (to a quite comparable degree). However, it was only in the deviant sound condition that the disruption persisted such that GDs were longer than was the case for the standard sounds. Because this effect occurred in GD, it suggests that the difference may have been driven by participants tending to refixate the post-target word (recall that the GD measure is the sum of fixations on a word before the eyes leave it). In line with this suggestion, we also observed an increased refixation rate on the post-target word in the presence of deviant sounds for the window condition compared with the no-window condition, whereas in the presence of a standard sound refixations rates were higher in the no-window condition. These results indicate that after a deviant sound relative to a standard sound, in the presence of a window, readers needed to refixate the post-target word to process it to a sufficient degree that the eyes might be moved forward to process new, upcoming information. It seems possible that the deviant sound had a longer lasting influence on post-target word processing because it was more distinctive (more deviant) than the standard sounds in relation to its tonal qualities.

The final aspect of our findings that we must discuss concerns the TVT results at the target and post-target words. At the target word, we observed interactive effects between sound and window. Readers spent more time reading in the absence of a window compared with when a window was present, and the difference between window conditions was largest in the presence of deviant relative to standard sounds, and larger in the presence of standard sounds relative to quiet. These increased total viewing times in the deviant condition as compared with the standard, and in the standard compared with the quiet, can be attributed to two (post hoc) possibilities; refixations or regressive fixations on the target. Note, though, that the TVTs at this region were only inflated by, on average, 61 ms relative to the GDs (mean GD = 333 ms; mean TVT = 394 ms). This means that readers fixated the target word during second pass reading only once in approximately every four trials; that is to say, readers did not often spend time revisiting the target word. Indeed, when we considered the probability of regression and refixation rates at the target word, we saw no significant interactions between sound and window for all conditions. That is, readers were no more likely to refixate the target word in first- or second-pass reading in the presence of a window compared with when no window was present, and this was true for all sound conditions. However, we must note that the probability of regression and refixation rate only reflect whether a reader was likely to make a regression/refixation (regardless of how many fixations were subsequently made). Furthermore, these measures do not provide insight into the time readers spent processing information. Therefore, it is possible that the interactive effects seen in TVT measures at the target word may be explained by an increase in the number, or simply a longer duration, of refixations (first-pass) or regressive (second-pass) fixations in the no-window compared with window condition for standard compared with quiet conditions, and in the presence of deviant compared with standard conditions. Furthermore, the overall pattern of effects that we see for TVT on the target word for standard and deviant sound conditions largely reflects the pattern of effects that we saw for the GD results at this region (and recall that the fixations that are included in the GD measure are also included in the TVT measure). Thus, it seems likely that the effects at this region are largely reflective of the effects that were observed during first pass reading of the target. That is, readers were more likely to make more, and/or longer, refixations in first pass reading of the target word when reading in the no-window condition. The only further noteworthy point concerns the TVT results for the quiet condition. Here, we observed quite comparable GD reading times under window (274 ms) and no-window (269 ms) reading conditions, but for TVT we observed somewhat longer times under no-window (360 ms) than window (339 ms) reading conditions. We suspect that these differences reflect readers’ tendency not to engage in extensive re-reading of sentences when they were presented under somewhat unusual visual reading conditions. Finally, there were no robust TVT effects at the post-target word and in line with our suggestion that participants did not engage in extensive re-reading in our experiment, as for the target word, there was only a modest 55 ms difference between average GD (289 ms) and average TVT (344 ms).

In summary, in this experiment we used the moving window paradigm to constrain parafoveal processing during reading with or without auditory distractors. We obtained four key findings. First, regardless of sound condition, we saw an overall effect of window at the sentence level in line with previous moving window studies. Second, the typical effect of window (reading times longer in window compared with no-window) was also seen at the local level in the quiet conditions. Third, sounds were more disruptive to reading as compared with quiet, and this disruption was increased when the sound was a deviant as compared with when it was standard. Finally, our results provide evidence that the presence of a sound (whether standard or deviant) influences aspects of parafoveal processing, such that readers engage in compensatory eye movements (i.e., shorter fixations) in the presence of background sound when reading is visually restricted. To conclude, the present results add to a growing body of literature using eye movement methodology to investigate distraction effects in reading, and such distraction effects can be attributed, at least in part, to disruption of parafoveal processing.

## Notes

1. The auditory deviant effect has also been shown to occur when deviants are presented within changing-state sequences (e.g., ABACA; Röer et al., 2014b) or when they are characterised by deviant inter-stimulus intervals or presented in a different voice to the standards (e.g., Hughes et al., 2005, 2007).

2. The most prominent competitor of the Duplex account of auditory distraction is the so-called “Unitary” (also termed “Single-process”) account which is heavily based on the Embedded-Process model (Cowan, 1999). These terms are used interchangeably within the literature but refer to the same account under which both deviant and changing-state (but not standard, steady-state) stimuli cause disruption by attentional capture. Within the literature, this account is sometimes referred to under a more general “attentional account,” which encompasses both the Unitary (Bell et al., 2010; Cowan, 2001) and graded attentional (Bell et al., 2019; Röer et al., 2014a; Schröger et al., 2000) accounts. Therefore, we make this distinction by using the more specific labels of “Unitary” and “Graded” for different aspects of the account in this article.

3. We adopt future tense in this manuscript up to the Results section, after which we adopt past tense, to demarcate the section of the manuscript that was pre-registered.

4. Data preprocessing for the Global Analyses was completed in the following order: (1) any participants with a comprehension accuracy lower than 80%, (2) trials with fixation durations shorter than 80 ms or longer than 800 ms using the “Perform 4-Stage Fixation Cleaning” function in DataViewer (SR Research), (3) observations with blinks occurring on the target or post-target words by filtering out fixations before/after blinks under “Data Filters” within Preferences in DataViewer (SR Research), (4) deviant trials where no deviant sound was presented (due to skipping the deviant word entirely, without returning), (5) deviant trials where the first target word(s) receiving standard sounds were skipped meaning the first sound that was played was a deviant sound, and (6) trials on which the sound was presented too early (i.e., before crossing the invisible boundary) or too late (after fixation onset).
